# The emerging role of echocardiography-based techniques in cardio-onco-hematology

**DOI:** 10.3389/fonc.2026.1696096

**Published:** 2026-01-28

**Authors:** Yichan Zhang, Mingxing Xie, Jing Wang, Bo Zhang

**Affiliations:** 1 Department of Ultrasound, China-Japan Friendship Hospital, Beijing, China; 2 Department of Ultrasound Medicine, Union Hospital, Tongji Medical College, Huazhong University of Science and Technology, Wuhan, China; 3 Clinical Research Center for Medical Imaging in Hubei Province, Wuhan, China; 4 Hubei Province Key Laboratory of Molecular Imaging, Wuhan, China; 5 National Center for Respiratory Medicine, State Key Laboratory of Respiratory Health and Multimorbidity, National Clinical Research Center for Respiratory Diseases, Institute of Respiratory Medicine, Chinese Academy of Medical Sciences, Department of Ultrasound, Center of Respiratory Medicine, China-Japan Friendship Hospital, Beijing, China

**Keywords:** cardiotoxicity, hematological malignancies, myocardial strain, myocardial work, stress echocardiography

## Abstract

In decades, the overall survival rate for cancer patients has improved significantly with continuous advances in cancer treatment technologies. However, cancer therapeutics-related cardiac dysfunction (CTRCD) has been one of the most worrying side effects, particularly in patients with hematological malignancies (HM), affecting the quality of life for cancer survivors. Early detection and prompt treatment are of vital importance. Echocardiography-based techniques can even identify subclinical myocardial damages, which is crucial for preventing irreversible myocardial damage. Conventional transthoracic echocardiography, speckle tracking imaging, myocardial work and stress echocardiography can assess myocardial global and segmental systolic function in multiple aspects. A significant decline of those parameters indicates that cardioprotective therapy should be initiated with close monitoring. This review will discuss the diagnosis and prognostic role of echocardiography-based techniques in patients with HM.

## 1 Introduction

Ranking as the ninth most common cause of death from cancer worldwide, the incident cases of hematological malignancies (HM) has been increasing for the past decade. Globally in 2020, It accounted for approximately 2.5% and 3.1% of all new cancer incidence and mortality, respectively (1–3). According to the Global Burden Disease (GBD) 2017 report, an estimated 141,317 new cases and 60,010 deaths of leukemia occurred in 2017 in China (4). In parallel, antitumor therapy has improved, leading to a significant increase in the overall survival of patients with HM. In 2030, leukemia mortality is projected to drop to 6.36/100000 among the males and 4.13/100000 among the females (4). Improved survival, however, had made cancer-related cardiovascular diseases (CVDs) increasingly prominent. Antitumor therapies, whether radiotherapy or chemotherapy, may lead to cardiovascular complications, including myocardial dysfunction, heart failure (HF), arterial hypertension, myocardial ischemia, cardiac rhythm disturbances, and QTc prolongation on electrocardiograms (5, 6). The rate of cardiovascular complications was markedly higher in patients with HM than other cancer patients, even after adjustment for cardiac risk factors (7, 8), making it challenging the prediction of long-term cardiovascular prognosis.

According to the 2021 consensus definition by the International Cardio-Oncology Society (ICOS), cancer therapy-related cardiac dysfunction (CTRCD) is defined as an adverse change in cardiac structure and/or function due to cancer therapy, which may present as asymptomatic cardiac dysfunction or symptomatic heart failure. Consequently, monitoring and managing CTRCD in clinical practice is critical for improving outcomes in cancer patients (9). Cardiotoxicity in HM arises from the interaction of three primary factors: chemotherapeutics, previous cardiovascular conditions and HM itself (10, 11). The current potentially cardiotoxic chemotherapeutic agents in HM are shown in **Table 1**, including anthracyclines (ANT), alkylating agents, antimetabolites, molecular-target agents, immune checkpoint inhibitors (ICIs), chimeric antigen receptor T therapy (CAR-T) and hematopoietic stem cell transplantation (HSCT) (12–19). The type, dose and mode of administration can affect the cardiovascular system, depending specifically on patient’s cardiovascular status. HM and CVDs share many of the same risk factors and patients with HM who preexist CVDs are at increased risk for cardiotoxicity when exposed cardiotoxicity chemotherapeutics (20, 21). Furthermore, HM is systemic disease, accompanied by an aberrant production of proinflammatory cytokines in particular tumor necrosis factor-α and interleukin-6, which may lead to immune-mediated myocardial depression and damage (22). Studies have been conducted to confirm that HM per se is associated with cardiac abnormalities before chemotherapy (22, 23).

**Table 1:** Common cardiotoxic drugs for hematologic malignancies and specific manifestations.

Types	Representative drugs	Applicable patients	Cardiotoxicity							
			Myocardial dysfunction	Heart failure	Hypertension	Myocardial ischemia	Arrhythmia	Pericarditis	Myocarditis	Others
Anthracyclines	Doxorubicin, daunorubicin, mitoxantrone	ALL, AML, Lymphoma	✓	✓			✓	✓	✓	
Alkylating agents	Cyclophosphamide	CLL, Lymphoma						✓	✓	Pulmonary hypertension
Antimetabolites	Cytarabine	AML, AML, CML, Lymphoma						✓	✓	
Tyrosine kinase inhibitors	Imatinib, Dasatinib, Nilotinib, Ponatinib	CML	✓	✓	✓	✓	✓			Arterial thrombosis
Immune checkpoint inhibitors	Ipilimumab, Nivolumab, Atezolizumab	Lymphoma		✓		✓	✓	✓	✓	cardiogenic shock
Immunomodulatory drugs	lenalidomide, thalidomide	MM				✓	✓			Arterial thrombosis
Proteasome inhibitors	Bortezomib, Carfilzomib, Ixazomib	MM		✓	✓	✓	✓			Cardiac arrest
Chimeric antigen receptor T therapy	/	B-ALL, MM, aggressive and indolent B-cell non-Hodgkin lymphoma	✓			✓	✓			sudden cardiac death
Hematopoietic stem cell transplantation	/	MM, Lymphoma, Hemoglobinopathies		✓			✓	✓	✓	Pulmonary hypertension

ALL, Acute Lymphocytic Leukemia; AML, Acute Myeloid Leukemia; CLL, Chronic Lymphocytic Leukemia; CML, Chronic Myeloid Leukemia; MM, Multiple myeloma; B-ALL, B-cell acute lymphoblastic leukaemia.

Echocardiography is crucial for the management of patients with CTRCD. Echocardiography-based techniques, such as speckle-tracking echocardiography (STE), myocardial work, and stress echocardiography, provide a quantitative measure of cardiac function. In this paper, we focus on echocardiography-based techniques, for the early diagnosis and prognosis of subclinical myocardial injury in HM patients treated with potential cardiotoxicity therapies.

## 2 Echocardiography-based techniques

### 2.1 Conventional transthoracic echocardiography

Transthoracic echocardiography remains the most widely used tool to detect CTRCD because of its noninvasive, radiation-free, and reliable nature compared to endomyocardial biopsy and radionuclide techniques. A position statement issued in 2020 stated that echocardiography is an important imaging method for assessing CTRCD (24). The definitions of cardiotoxicity are based on a reduction in left ventricular ejection fraction (LVEF), a reduction of which >10% from baseline falling below the lower limit of normal considered as 50%, on the basis of the 2022 ESC Cardio-Oncology (9). Right ventricular (RV) systolic function parameters, such as RV fractional area change (FAC), tricuspid annular plane systolic excursion (TAPSE), were considered to be associated with the morbidity and mortality (25, 26).

### 2.2 Speckle tracking echocardiography

And yet reduced LVEF has been demonstrated to be a late manifestation of CTRCD. Studies showed that although therapeutic interventions were initiated after a significant decline in LVEF, systolic function was not recovered in over 58% of patients (27). In recent years, speckle tracking echocardiography (STE) has been developed as a new imaging tool for evaluating cardiac function. STE is a rapid, highly feasible, and easy-to-perform technique for estimating cardiac function. In addition to assessing myocardial motion along the ultrasound beam, STE can also quantify myocardial torsion, circumferential strain, and radial motion (28). Subclinical cardiac dysfunction in cancer patients was defined by 2020 ESMO consensus as an absolute decrease from baseline in LV global longitudinal strain (LVGLS) by STE of ≥5% or a relative decrease from baseline in the LVGLS of ≥12% or Troponins elevation from baseline (5).

**Figure 1:**
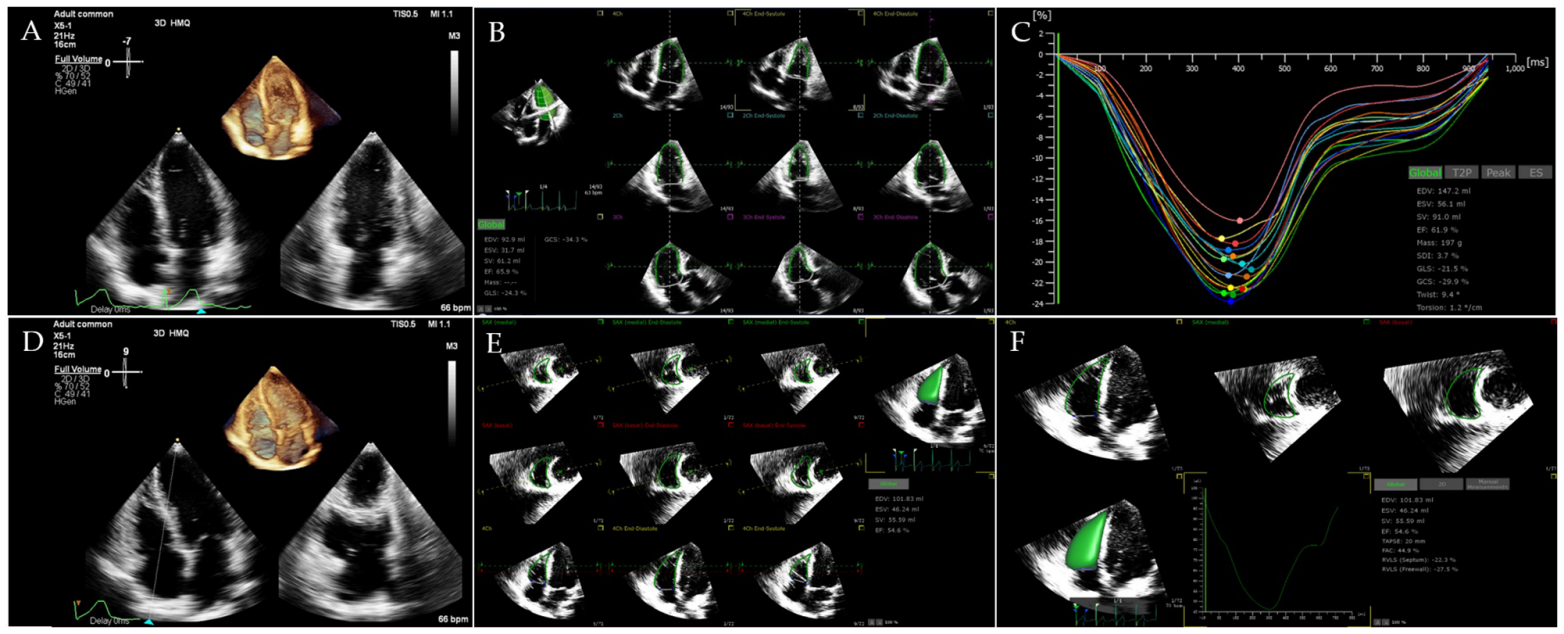
Ventricular three-dimensional image. **(A)** Three-dimensional image of LV-focused apical 4-chamber view. **(B)** LV endocardial border identification and tracking at end-systole and end-diastole. **(C)** Longitudinal strain of LV. **(D)** Three-dimensional image of RV-focused apical 4-chamber view. **(E)** RV endocardial border identification and tracking at end-systole and end-diastole. **(F)** Longitudinal strain of RV free wall and RV volume-time curve were automatically generated.

STE and real-time 3D echocardiography allow clinicians to precisely quantify RV structure and function and to detect subclinical RV dysfunction early, thereby providing comprehensive diagnostic and prognostic information for patients with a variety of cardiovascular diseases (29–33). More recently, RV free wall longitudinal strain (RVFWLS), a more accurate and sensitive tool for assessing RV systolic function, has been recognized to provide superior prognostic value over RVGLS as well as conventional echocardiographic parameters in other cardiovascular diseases (29, 33–39). **Figure 1** shows the three-dimensional STE analysis for the LV and RV.

### 2.3 Myocardial work

**Figure 2:**
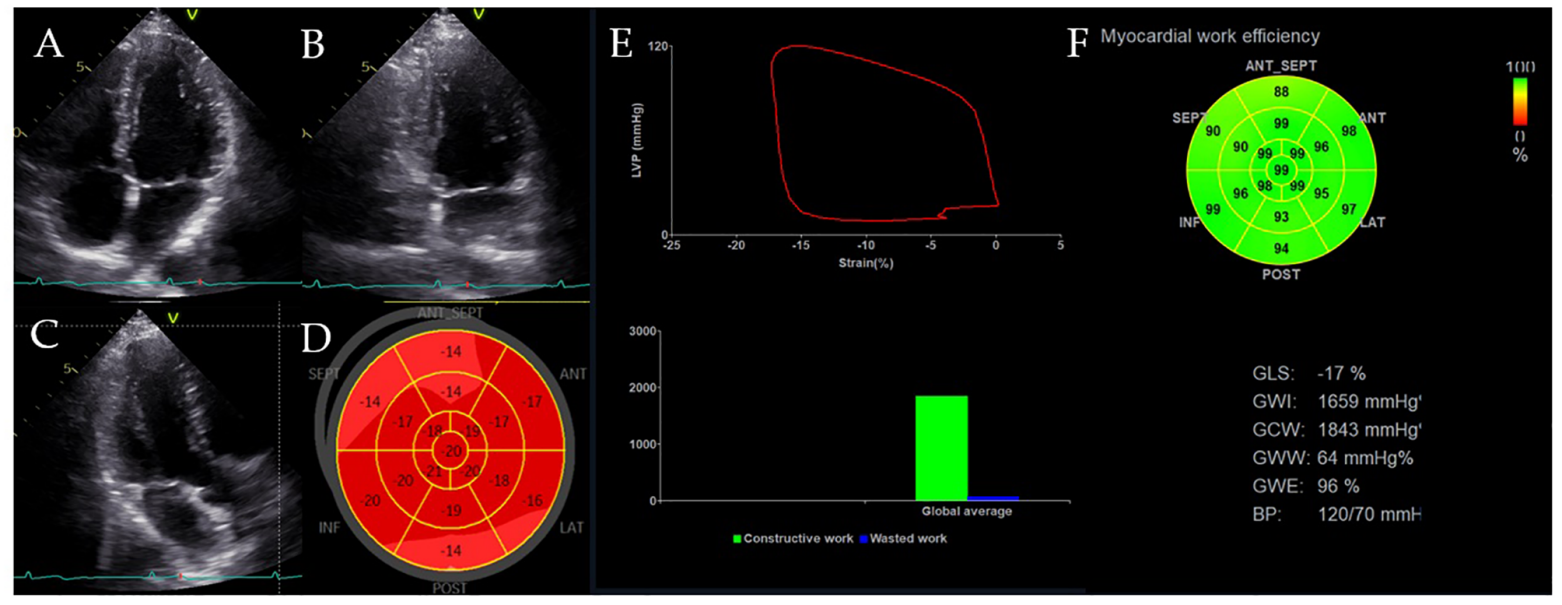
LV pressure-strain loop and MW indices. **(A–C)** LV-focused apical 4-chamber, 2-chamber and 3-chamber view. **(D)** Bull’s eye view of LVGLS. **(E, F)** Pressure-strain loop and global constructive work, global wasted work and global work efficiency.

Myocardial work (MW) is a novel marker of LV systolic function that can be used to noninvasively determine total active myocardial performance (40). Myocardial work (MW) comprises four components: global work index (GWI), global constructive work (GCW), global wasted work (GWW), and global work efficiency (GWE). MW has benefits over LVEF and GLS as it includes the afterload-dependent limitation and dynamic myocardial contraction in relationship to various loading conditions (41). To date, the technique has been investigated for assessing myocardial ischemia, valvular heart diseases, hypertrophic cardiomyopathy, oncological cardiology (42–45), indicating its incremental value relative to GLS. **Figure 2** shows the myocardial work analysis for the RV.

### 2.4 Stress echocardiography

Stress echocardiography (SE) is a reliable and well-established cardiac imaging modality, providing a dynamic assessment of global and regional LV function under conditions of physiologic or pharmacologic stress. It was initially used for assessing coronary artery disease (46). In recent years, there has also been an increasing use of SE in the assessment of non-ischaemic cardiac disease, given its ability to assess functional capacity and hemodynamic changes during exercise, which can help guide therapy and inform prognosis (47). In cardio-oncology, anticancer drugs can impair cardiac function through multiple mechanisms: a direct toxic effect on the myocardium (reducing LV reserve) or by favoring myocardial ischemia via accelerated atherosclerosis, endothelial dysfunction, or vasospasm (9, 48). These subtle changes often occur under stress and are not detectable by conventional echocardiography, which can only assess cardiac pump function at rest. Therefore, techniques like stress echocardiography are needed to compensate for these shortcomings.

## 3 Diagnostic and prognosis value of echocardiography

### 3.1 Global function of the left ventricle

LV dysfunction is often present in patients with HM and HM itself is associated with cardiac alterations (49). Bruna et al. (22) compared the LV structure and function in 76 patients with acute leukemia (AL) before initiation of chemotherapy and 76 patients without cancer matched for age, gender, hypertension, and the presence of diabetes. They revealed that despite similar LVEF, patients with AL had higher LV mass and volumes and lower LVGLS than patients without cancer. Compared to 28 women with breast cancer, those with AL had significantly lower LVGLS. Furthermore, low LVEF, high body mass index, and low circulating lymphocyte count were all independently associated with reduced GLS. This suggests that the absolute number of circulating lymphocytes plays a significant role in the myocardial changes observed in AL patients. Additionally, studies have shown that baseline LVGLS is a strong predictor of late-onset heart failure in survivors of HM (23). In a study of breast cancer patients undergoing chemotherapy, Lorenzini et al. confirmed that 3D-LVEF could detect CTRCD earlier and more reliably, with lower intero-bserver variability (50). The effectiveness of combining 3D-LVEF and GLS for early detection of cardiotoxicity in patients with HM warrants further investigation.

The strategy for the early detection of cardiotoxicity involves serial echocardiographic imaging to identify LV dysfunction. However, changes in systolic blood pressure (SBP) between echocardiographic visits may confound the diagnosis of CTRCD. Kosmala at al (51). investigated the incremental value of MW indices in diagnosing and prognosticating CTRCD by dividing those with CTRCD into subsets with and without a follow-up SBP increment >20 mmHg (CTRCD+BP+ and CTRCD+BP−). Patients from the CTRCD+BP− group demonstrated significantly larger decreases in the GWI and GCW than the CTRCD+BP+ groups, which may indicate some degree of LV contractility reserve in response to the elevation of LV afterload. The other MW parameters did not differ. From a practical point of view, the evaluation of MW may be a useful adjunct to GLS in patients with significant BP fluctuations during follow-up.

One of the principal limitations of established methods for detecting CTRCD is that they evaluate cardiac function at rest, whereas the symptoms of heart failure are usually present during exertion. Cardiac reserve— defined as the increase in cardiac function from rest to peak exercise— must be assessed under the hemodynamic and metabolic demands of exercise to provide a more complete determination of the heart’s functional capacity (52). Previous studies (53) found that anthracycline-based adult acute lymphoblastic leukemia (ALL) survivors at a median of 21 years off treatment had subclinical cardiac dysfunction identified through stress echocardiography. Among these asymptomatic patients, EF at peak exercise was significantly lower in the 23 anthracycline-exposed survivors than in the 12 healthy controls, and that 10 of the patients reduced their EF at stress compared to EF at rest. These results suggested that echocardiographic evaluation at peak exercise could reveal otherwise undiagnosed impaired systolic function (54–56).

It is widely accepted that LV diastolic dysfunction is common among cancer patients. Global diastolic strain parameters derived from STE have been proposed as new measures of elevated LV filling pressures because they are less angle-dependent and can directly evaluate ventricular relaxation (57, 58). Yet no clear evidence has shown that it can predict future LV systolic dysfunction in HM patients. Hochstadt et al. (59) evaluated the correlation between a novel parameter— diastolic strain slope (Dss) and routine echocardiography diastolic parameters in patients receiving cancer therapy with minority HM patients. The results showed that Dss was significantly correlated with e’ average. Furthermore, the change in mid-segment Dss between the first and third echocardiograms was significantly associated with the development of systolic dysfunction. Owing to limited data, diastolic strain is not currently part of the routine STE evaluation in the HM population.

### 3.2 Segmental function of the left ventricle

Although conventional echocardiography is the most commonly used imaging modality in the clinical evaluation and diagnosis of CTRCD, it is limited to the overall functional assessment. STE can provide information on the local myocardial deformation of LV, RV and atrial wall, allowing a multi-parameter accurate assessment of LV dysfunction.

A retrospective study (60) included 60 patients with newly diagnosed diffuse large B-cell lymphoma (DLBCL) treated with R-CHOP chemotherapy. 3D-GLS, 3D-GCS and 3D LV longitudinal strain of different LV segments (LS) were measured by 3D-STE at baseline, after the completion of two cycles and four cycles of the regimen respectively. Compared with baseline, 3D-GLS reduced significantly after four cycles of anthracycline therapy, while 3D-GCS showed no significant variation during the whole procedure. However, abnormalities in segmental myocardial strain are already present even early. LS of apical anterior and septal walls decreased significantly after two cycles of chemotherapy. This study demonstrated that anterior and septal walls of the LV are more sensitive to drug toxicity and the development of cardiotoxicity may start from apical segment to basal.

In another study, a multi-layer strain approach was used to analyze layer-specific ventricular deformation. This approach revealed a reduction in subendocardial circumferential strain and the transmural circumferential gradient in long-term survivors of lymphoma (61). The endocardium is more vulnerable because of its higher energy requirements. STE allows identification of segmental LV wall impairment and can reveal the progression of dysfunction following anthracycline chemotherapy, thereby providing more reliable information for monitoring disease progression (62). However, the value of layer-specific strain is considered limited and contradictory, primarily due to poor endocardial border definition which compromises tracking accuracy (63).

### 3.3 Global function of the left atrium

The left atrium (LA) plays an important role in modulating left ventricular filling, contributing up to a third of cardiac output. LA function is complex, comprising of three main phases: (1) the reservoir phase during systole when the chamber fills with blood; (2) the conduit phase during early diastole, which corresponds to passive ventricular filling; and (3) the active contractile (booster) phase in late diastole. LA functional indices, including LA volumes, have been reported to be associated with LV function and have prognostic value in cardiovascular disease (64–66). Menon et al. (67) compared LA volumes in anthracycline-treated survivors (most with HM) to normal controls. LA reservoir and conduit function parameters were significantly lower in controls. The authors speculated that these findings might reflect early changes in LA compliance associated with anthracycline exposure. They concluded that assessment of LA volumes and function as prognostic markers of cardiotoxicity in this population is warranted.

LA strain has been shown to correlate with the degree of diastolic dysfunction (68). Cardiotoxic chemotherapy has been associated with the development of diastolic dysfunction, which often precedes LV systolic dysfunction. Moreover, the progression to clinical heart failure with preserved ejection fraction (HFpEF) is associated with a long-term mortality rate similar to that of heart failure with reduced ejection fraction (HFrEF) (69). A study by Emerson et al. (70) reported similar findings. The study assessed 79 HSCT survivors with prior anthracycline exposure and compared them to 79 normal controls. LV systolic dysfunction with reduced LVEF (13/79) or GLS (29/79) was present in the transplant group. Both LA reservoir strain (which modestly correlated with mitral annular e′ velocity) and LA conduit strain were reduced in the HSCT survivors compared to controls. Using current diastolic function guidelines, 26/79 patients had evidence of diastolic dysfunction. However, using LA reservoir strain identified an additional 35 patients. Thus, LA strain can identify early diastolic dysfunction in HSCT survivors, prompting increased surveillance and treatment.

### 3.4 Global function of the right ventricle

In a retrospective analysis (71) that involved 274 adult lymphoma survivors who underwent autologous HSCT before, investigators evaluated TAPSE, FAC, RV index of myocardial performance (RIMP) and RVGLS in these patients. All parameters of RV systolic function were impaired in lymphoma survivors compared with control subjects. Furthermore, a greater cardiotoxic treatment burden was associated with larger RV functional impairment. Survivors of allogeneic HSCT are at higher risk of cardiovascular disease, but the occurrence of RV dysfunction was less frequent and milder than coexisting LV dysfunction in this cohort (72, 73). The role of SE in the assessment of RV systolic and diastolic function is not yet established (74–76), and consequently, current guidelines do not endorse its use for surveillance.

### 3.5 Segmental function of the right ventricle

The published guideline recommends a normal reference value only for RVFWLS. In contrast, it does not provide a normal value for RVGLS, which is the average strain of six myocardial segments. The accuracy of RVGLS may be confounded by left ventricular (LV) systolic function because the interventricular septum—conventionally considered part of the LV—is included in its calculation (77). Furthermore, a reduction in RVFWLS was independently associated with anthracycline-related RV systolic dysfunction (78).Due to the paucity of data, the definition of RV cardiotoxicity is not completely established in cardio-oncology. Zhao et al. (79) defined RV cardiotoxicity in a cohort of 74 patients with DLBCL treated with anthracycline as either a >10% relative reduction in RVEF measured by 3D echocardiography, or a relative reduction of >5% to an absolute value of <45%. They confirmed that ΔRVESV and ΔRVFWLS between baseline and after the completion of 4 cycles were associated with subsequent RV cardiotoxicity. Moreover, a relative decrease in ΔRVFWLS of >12.4% and a relative increase in ΔRVESV of >13.2% were able to discriminate between patients with and without RV cardiotoxicity. These findings are significant as they provide early data to support the potential use of 3DE-derived RV parameters in cardio-oncology.

## 4 Clinical management on myocardial injury

For all patients scheduled to receive cancer therapies with a clinically significant risk of cardiotoxicity, a baseline cardiovascular risk assessment is recommended. For patients identified as high or very high risk, referral to a cardio-oncology service is advised to initiate risk mitigation strategies (80). There are two potential responses to myocardial injury: initiation of cardioprotective medications and modifying or interrupting cardiotoxic therapy. The latter may have a negative effect in cancer treatment (81). Numerous drugs have been used as cardioprotective care. Dexrazoxane, an FDA-approved cardioprotective drug, has been successfully used to ameliorate cardiac toxicity seen in anthracycline-based (e.g., doxorubicin, daunorubicin, epirubicin) chemotherapy recipients for cancer. It is only recommended for use intravenously of choice to decrease the incidence of cardiomyopathy and congestive heart failure (82). In the study by Asselin et al. (83), 264 patients who received doxorubicin alone were compared with 273 who received doxorubicin plus dexrazoxane. The doxorubicin-alone group had worse mean LV fractional shortening, wall thickness, and thickness-to-dimension ratio z scores measured 3 years after diagnosis. Their mean fractional shortening z scores remained diminished when measured 3.5 to 6.4 years after diagnosis. In parallel, the rates of infection, hematologic toxicity, and central nervous system toxicity did not differ between two groups. This study showed that dexrazoxane reduced the risk of clinical heart failure and cardiac events in HM patients undergoing anthracycline chemotherapy without significantly impacting cancer outcomes (84–86). In addition, a number of medications**—** angiotensin converting enzyme inhibitors, angiotensin receptor blockers, beta-adrenoceptor blockers and statins**—** have proven benefit in the prevention of cardiotoxicity (87–91).

## 5 Conclusions

Prevention is better than cure, hence the necessity to use high sensitivity imaging methods to detect early, subclinical changes and allow prompt intervention to prevent further damages. Echocardiography-based techniques can offer important clinical and prognosis information in HM and can provide a more comprehensive and detailed evaluation of ventricular systolic and diastolic function, allowing for an early detection of subclinical myocardial injury. One of the challenges in the field oncological cardiology lies in the lack of universally adopted, granular definitions. While the ESMO consensus provides a broader clinical framework, the ICOS/ESC definitions offer a more precise, imaging-based threshold for CTRCD (5, 9, 80). Comparative studies analyzing outcomes based on different definitions are a needed area of future research. A united effort by oncologists and cardiologists is needed to develop guidelines for the diagnosis and management of chemotherapy-related cardiotoxicity.
